# Impact of Family Planning Program on Related Drug Consumption in Iran: A Ten-Year Trend Analysis (2014 - 2023)

**DOI:** 10.5812/ijpr-164664

**Published:** 2026-06-21

**Authors:** Matin Jafari

**Affiliations:** 1 Faculty of Pharmacy, Tehran University of Medical Sciences, Tehran, Iran

**Keywords:** Drug Consumption, Population Policy, Impact Assessment

## Abstract

**Background:**

Global demographic patterns have shifted substantially over the past century, with declining fertility and accelerated population aging emerging as dominant trends. Iran exemplifies this transition, with an anticipated population peak around 2050 followed by sustained decline. In response, the Family Support and Population Rejuvenation Law (2021) reversed earlier fertility-limiting policies by expanding insurance coverage for infertility therapies while restricting access to subsidized contraceptives and abortifacients, thereby positioning the pharmaceutical sector as a key policy instrument.

**Objectives:**

This study aimed to evaluate the impact of the 2021 law on national consumption patterns of reproductive health-related medicines in Iran from 2014 to 2023, including fertility agents, contraceptives, abortifacients, and neonatal treatments.

**Methods:**

A retrospective time-series drug-utilization study was conducted using national wholesale data for 52 reproductive medicines, standardized according to the World Health Organization Anatomical Therapeutic Chemical and Defined Daily Dose methodology. Monthly and annual utilization was expressed as defined daily doses per 1000 inhabitants per day. Segmented linear regression and paired t tests were used to compare consumption trends before and after implementation of the law.

**Results:**

Of the 52 products examined, 19 dosage forms (37%) across 5 Anatomical Therapeutic Chemical codes showed significant level or slope changes after the law (P < 0.05). Use of infertility treatments, particularly recombinant follitropins, menotropins, and chorionic gonadotropins, increased markedly (+48%, P = 0.01). Despite legal restrictions, use of prostaglandin abortifacients, such as misoprostol and carboprost, also increased (+19%, P = 0.047). Total contraceptive use remained stable; however, market share shifted toward newer low-dose combinations, suggesting substitution rather than reduction. Neonatal surfactant use more than doubled after the law (P < 0.001), raising concerns about increased premature births amid limited prenatal screening.

**Conclusions:**

Although the Family Support and Population Rejuvenation Law expanded access to infertility medicines, it did not reduce contraceptive or abortifacient use. Shifts in consumption patterns suggest that regulatory restrictions alone may result in unintended consequences, including clandestine abortion and neonatal complications. A more balanced, evidence-based policy approach is essential to align demographic goals with maternal and newborn health.

## 1. Background

The world’s population has increased from approximately 400 million in 1500 to more than 8 billion in the early 21st century, with United Nations projections indicating a further rise to approximately 9.7 billion by 2050 (1, 2). Widespread family-planning campaigns initiated in the 1960s successfully reduced fertility but also reshaped global medication-consumption patterns related to contraception and reproduction (3). By 2025, more than 66% of the global population lived in countries with sub-replacement fertility (2). Projections indicate that the number of people aged 60 years or older will reach 1 billion by 2030 and 1.6 billion by 2050, while the cohort younger than 20 years will remain nearly static.

**Figure 1.**
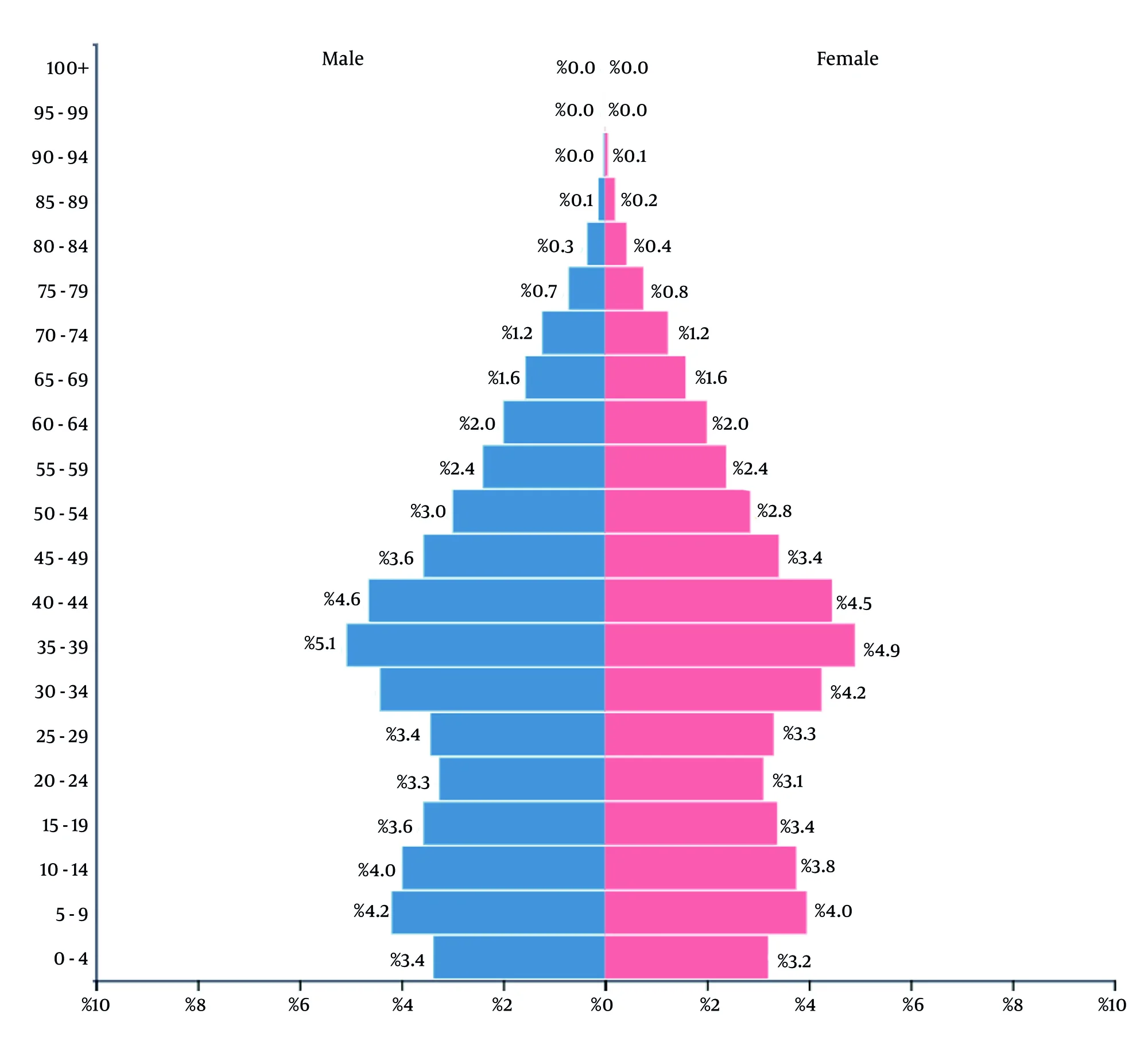
Population pyramid of Iran (Islamic Republic of), 2024.

Iran mirrors these global demographic trends, with a current population exceeding 86 million. The proportion of Iranians aged 65 years or older is expected to triple between 2023 and 2049, representing a rate of population aging that substantially exceeds that of France, the United Kingdom, and the United States. Figure 1 illustrates Iran’s population pyramid, and Table 1 summarizes key demographic indicators relevant to this transition (4).

**Table 1.** Key Demographic Indicators for Iran

Indicators	Latest/Projected Value
**Total population, estimated**	≈86 million
**Total fertility rate**	1.7
**Median age**	34
**Population aged ≥ 65 y**	≈7.4%
**Projected population peak**	≈92 million, by 2050
**Aging pace**	Faster than France, the United Kingdom, and the United States

In response to these demographic challenges, Iran has implemented several policy measures. In 2009, the Family Planning and Population Law was amended to modify restrictions on fourth children in households. Subsequently, various supportive measures for childbearing were implemented, culminating in the Family Support and Population Rejuvenation Law in October 2021.

Adopted in 2021, Iran’s Family Support and Population Rejuvenation Law shifted national family-planning policy from fertility limitation to fertility promotion by expanding insurance coverage for infertility therapies. The law directly regulates pharmaceutical access, for example, by banning the free or subsidized distribution of contraceptives without a prescription and expanding insurance coverage for infertility treatments. This positioning makes the pharmaceutical supply chain a central mechanism of population policy.

Assessing how medication-consumption patterns evolve in response to such legislative shifts is essential for evidence-based population health policy. It is also critical for balancing pro-natalist goals with the protection of maternal and neonatal health. However, despite substantial debate regarding the law’s implications, no national time-series evidence has documented how reproductive-health pharmaceutical utilization changed before versus after the legislation.

Cross-country drug-utilization research indicates that regulatory levers can rapidly reshape medicine volumes and mixes (5, 6). For reproductive health products, policy changes have produced measurable effects on fertility outcomes (7, 8). However, comparative evaluations conclude that economic incentives, such as parental leave and childcare subsidies, rather than pharmaceutical restrictions, are the dominant policy tools in most Organisation for Economic Co-operation and Development countries (9, 10). In Iran, recent qualitative analyses and local cross-sectional studies describe the law’s simultaneous expansion of infertility subsidies and tightening of contraceptive access but provide no quantitative time series of downstream pharmaceutical use (6, 11).

## 2. Objectives

This study aimed to evaluate the impact of the Family Support and Population Rejuvenation Law on annual consumption patterns of reproductive-health-related medicines in Iran over a 10-year period (2014 - 2023), expressed in defined daily doses per 1000 inhabitants per day.

## 3. Methods

### 3.1. Study Design and Setting

A retrospective, quantitative drug-utilization study with a time-series design was conducted over a 10-year period from March 2014 to March 2024. The unit of analysis comprised national-level monthly sales data from 2021 to 2024 and annual sales data from 2014 to 2021 for selected reproductive-health medicines in Iran.

### 3.2. Selection of Study Medicines

Candidate substances were identified based on the provisions of the law and coded according to the World Health Organization Anatomical Therapeutic Chemical (ATC) classification system. A 2-round expert survey involving gynecologists and clinical pharmacists was conducted using Google Forms (Google LLC, USA) to validate and refine the list. Content validity was confirmed, with a content validity index greater than 0.8; internal consistency was strong, with Cronbach α = 0.82; and test-retest reliability over a 2-week interval was acceptable, with an intraclass correlation coefficient of 0.86. Following consensus, a final list of 52 medicinal products was established, and annual defined daily dose per 1000 inhabitants per day values for each medicine from 2014 to 2023 were incorporated into the Supplementary Table to ensure full transparency of the underlying utilization trends.

The defined daily dose is the assumed average maintenance dose per day for a drug used for its main indication in adults. In the ATC classification system, active substances are divided into groups according to the organ or system on which they act and their therapeutic, pharmacological, and chemical properties. Drugs are classified into 5 different levels.

### 3.3. Data Extraction, Cleaning, and Validation

Package-level wholesale volumes and factory prices were extracted from the Iranian Drug Statistical Yearbooks and the accompanying Monthly Pharmaceutical Bulletins issued by the Iran Food and Drug Administration, which constitute the official repository of national medicine-distribution data.

A systematic 3-step procedure was implemented to ensure data quality.

Step 1: Retrieval. Data were extracted from the Iran Food and Drug Administration National Drug Statistical Yearbooks and Monthly Pharmaceutical Bulletins.

Step 2: Validation and cleaning. Pack sizes were validated against the Integrated Registration Code database to ensure uniform conversion from packs to dosage units. To detect reporting errors, a price-volume coherence check was performed. Unit prices, expressed as rial per unit, were recomputed, and observations deviating by ± 20% from the reference ex-factory tariff were flagged. Miscoded Integrated Registration Codes were manually corrected to prevent artificial spikes or drops in volume.

Step 3: Standardization. Each product was linked to the World Health Organization ATC/defined daily dose index. World Health Organization guidance for complex dosing was applied by adjusting oral contraceptive defined daily doses to account for pill-free intervals (eg, 21-day packs = 0.75 defined daily doses) and by standardizing neonatal surfactants based on a 1.3-kg average birth weight for ATC R07AA. Products without an official defined daily dose were excluded; none were present in the final list.

### 3.4. Measurement of Utilization

Population-standardized utilization was calculated as defined daily doses per 1000 inhabitants per day. This metric estimates how many individuals per 1000 people were potentially receiving a standard daily dose of the medication. The formula used was:

$$\mathrm{DID}=\frac{S\times \mathrm{Strenght}\times 1000}{\mathrm{DDD}\times 365\times P}$$

In this formula, S is the number of medications used, Strength is the amount of active ingredient per unit in World Health Organization defined daily dose units, and P is the mid-year resident population published by the Statistical Center of Iran.

### 3.5. Statistical Analysis

All analyses were conducted using SPSS.

Descriptive statistics were used to summarize trends in defined daily dose per 1000 inhabitants per day for each ATC code.

Segmented linear regression using an interrupted time-series design was applied to estimate the impact of the 2021 law. The model evaluated 3 parameters: the preintervention trend, represented by slope β1; the immediate level change after policy implementation, represented by the change in intercept; and the postintervention change in trend, represented by the change in slope β3. The change point was strictly defined as the year of law enactment, 2021.

Because the law was implemented midyear, 2021 in the Iranian calendar was considered a transition period. Accordingly, this year was excluded from prelaw and postlaw mean comparisons to avoid misclassification of exposure but was retained as the intervention point in the interrupted time-series analyses. Potential policy-anticipation effects were considered, and short-term supply fluctuations were examined descriptively to avoid obscuring genuine supply-chain variation.

To further assess the impact of the law, paired t tests were used to compare mean defined daily dose per 1000 inhabitants per day between the prelaw period, 2014 to 2020, and the postlaw period, 2021 to 2023, in the Iranian calendar, alongside change-in-slope estimates derived from the segmented regression models.

The longer preintervention period enabled stable estimation of baseline trends, whereas the shorter postintervention period reflected data availability following recent policy implementation and limited long-term inference.

### 3.6. Ethical Considerations

The study used secondary, aggregate sales data with no patient identifiers; therefore, ethics committee review was waived under national regulations.

## 4. Results

Of the 52 commercial dosage forms analyzed, 33 dosage forms (63%) and 10 ATC codes (67%) showed no statistically significant change in the mean defined daily dose per 1000 inhabitants per day or in trend after enactment of the law. Conversely, 19 dosage forms (37%) across 5 ATC codes exhibited significant level and/or slope shifts (P < 0.05). These findings indicate that, although the law did not uniformly affect the overall reproductive pharmaceutical market, it triggered selective and significant increases in specific therapeutic categories.

### 4.1. Prostaglandins (ATC G02AD)

**Figure 2.**
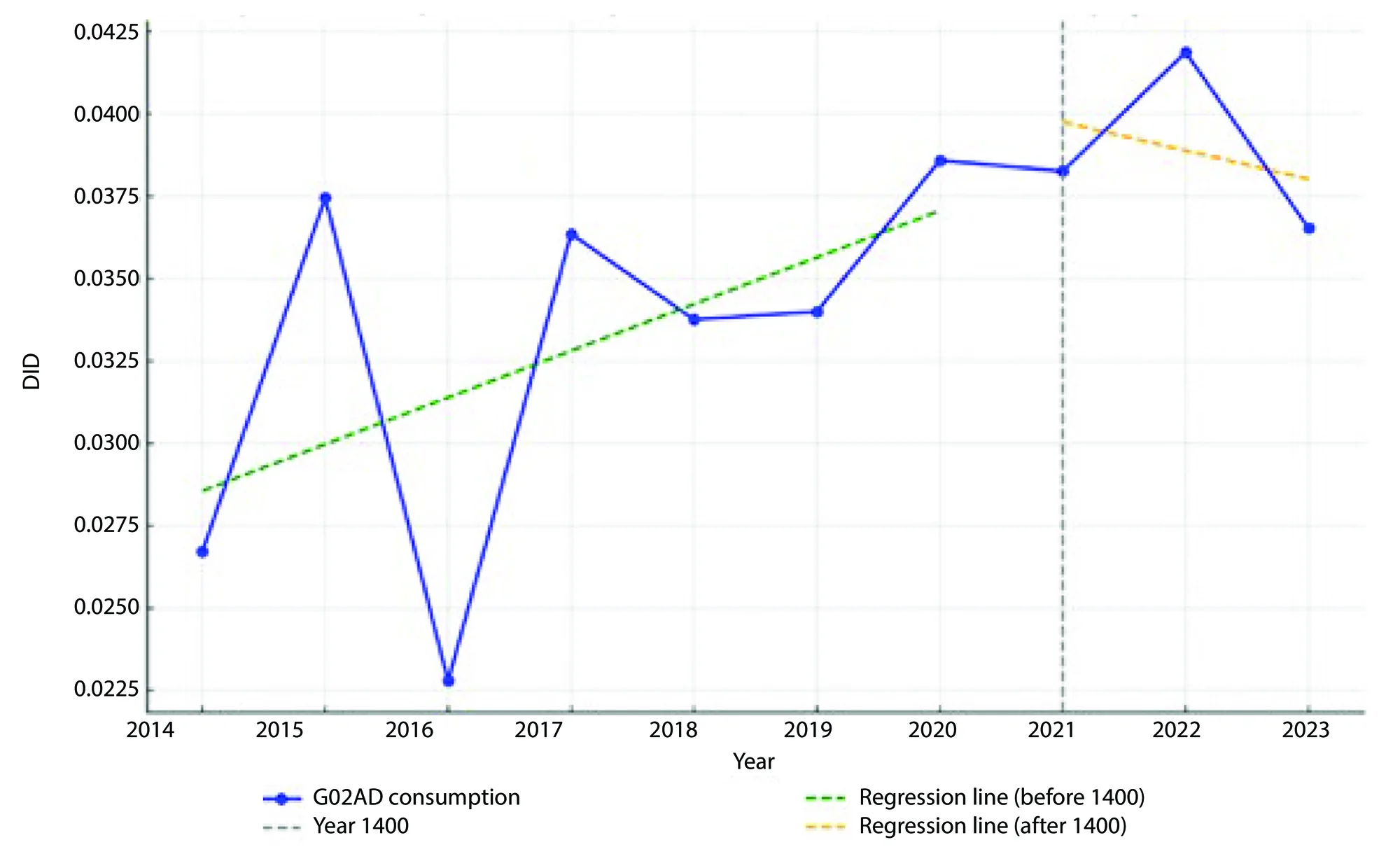
Annual procurement and consumption trends for prostaglandins.

Annual procurement and prelaw and postlaw changes are shown in Figure 2. Mean utilization of prostaglandin abortifacients, including misoprostol and carboprost, increased significantly from 0.033 defined daily doses per 1000 inhabitants per day in 2014 to 2021 to 0.039 defined daily doses per 1000 inhabitants per day in 2022 to 2023, representing a 19% relative increase (P = 0.047). Although mean utilization increased, the slope of the consumption trend shifted from +0.0001 to -0.0009 after 2021. However, this trend change did not reach statistical significance (P = 0.412), suggesting that the immediate postlaw increase may have been a temporary level shift rather than a sustained change.

### 4.2. Systemic Contraceptives (ATC G03A/G03AA/G03AC)

*Aggregate* oral and injectable contraceptive consumption continued its long-standing decline, with no significant change in the mean defined daily dose per 1000 inhabitants per day after enactment of the law. However, molecule-level analysis revealed notable heterogeneity. Although older monophasic combinations and medroxyprogesterone injections continued to decline, newer generations, specifically drospirenone, chlormadinone, and dienogest combinations, showed steeper upward trends in the postlaw period. This pattern suggests a shift toward newer pharmaceutical generations despite the generally restrictive policy.

Older low-dose monophasic combinations, including ethinyl estradiol plus norgestrel and ethinylestradiol plus levonorgestrel, and medroxyprogesterone injections remained in decline.

Newer contraceptive combinations, including drospirenone plus ethinyl estradiol, chlormadinone plus ethinyl estradiol, and dienogest plus ethinyl estradiol, showed a steeper postlaw increase, partially offsetting the overall contraction.

**Figure 3.**
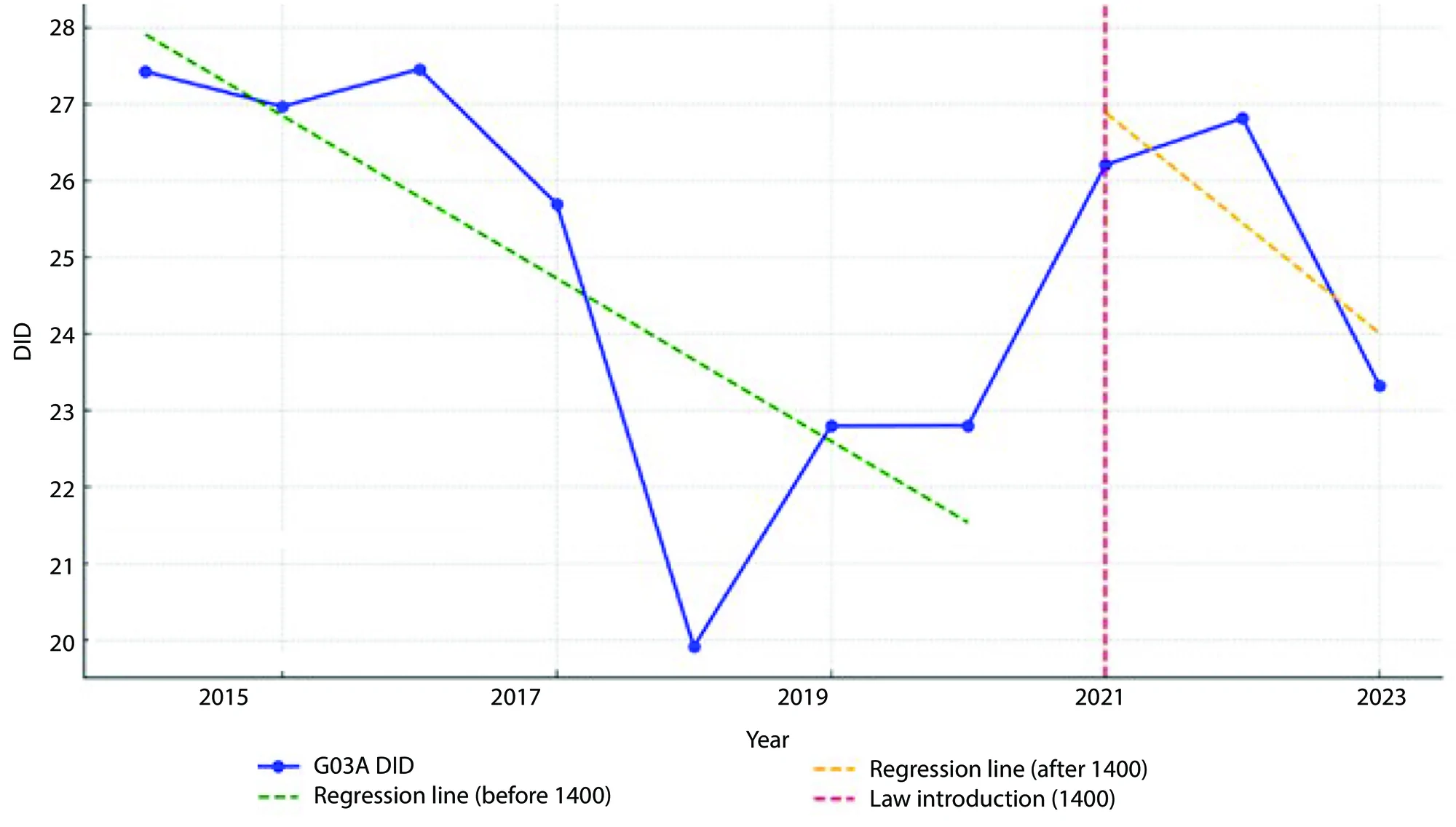
*Aggregate contraceptive* procurement and consumption trends.

*Aggregate contraceptive* procurement and consumption trends are shown in Figure 3.

### 4.3. Infertility Therapies

#### 4.3.1. Gonadotropins and Antigonadotropins (ATC G03G, G03GA)

**Figure 4.**
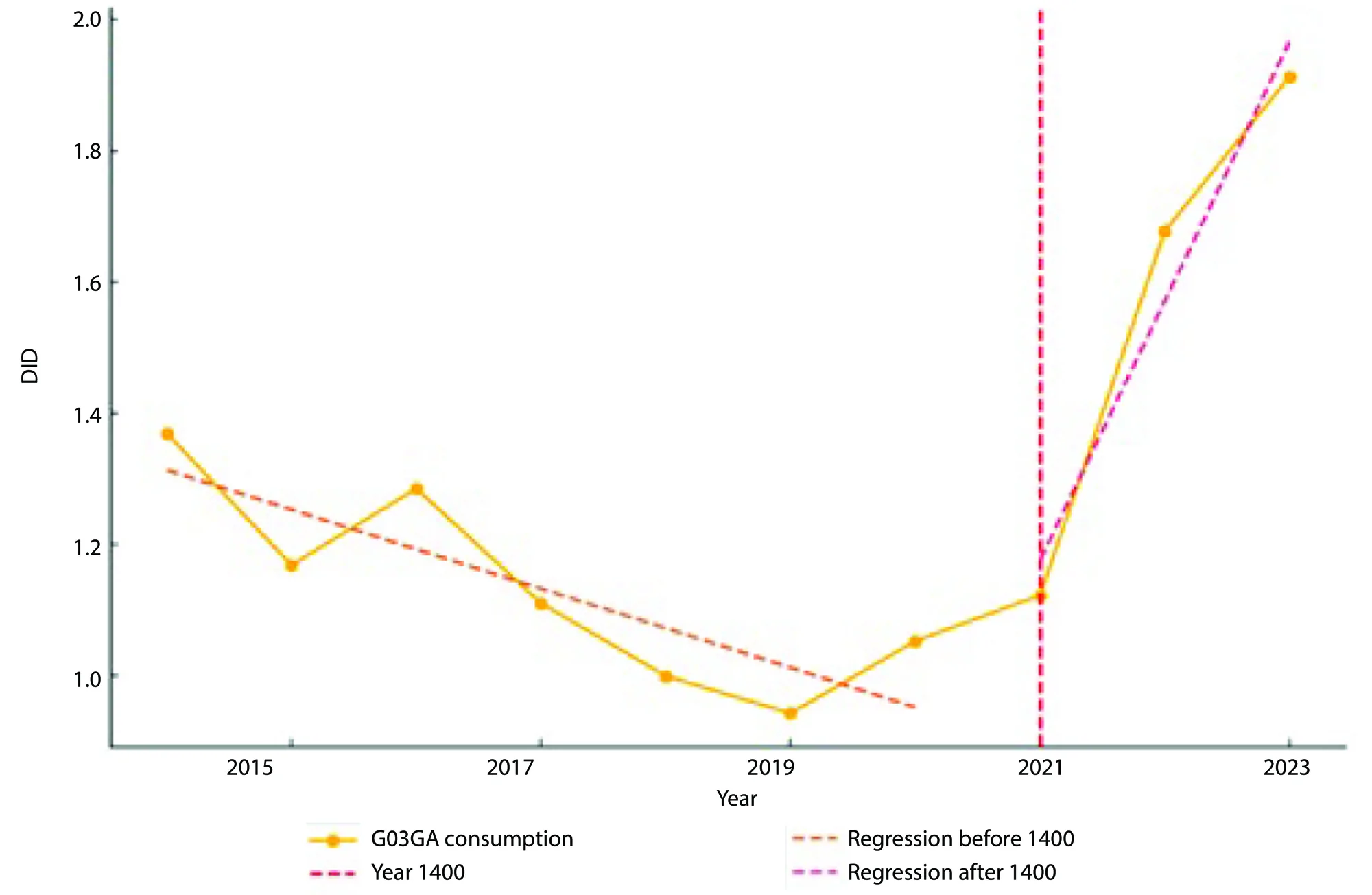
Annual purchasing and consumption trends for gonadotropins.

Annual purchasing and consumption trends for gonadotropins are shown in Figure 4. This class showed the largest absolute growth. The mean defined daily dose per 1000 inhabitants per day increased by 48% after enactment of the law (P = 0.01), with segmented regression confirming a significant trend reversal from -0.007 to +0.032 defined daily dose per 1000 inhabitants per day per year. This increase was primarily driven by follitropin alfa (β +0.014), follitropin beta (β +0.011), menotropins (β +0.008), and corifollitropin alfa (β +0.005). This surge is directly consistent with the law’s mandate to expand insurance coverage for infertility treatments.

#### 4.3.2. Antigonadotropins (ATC H01CC)

**Figure 5.**
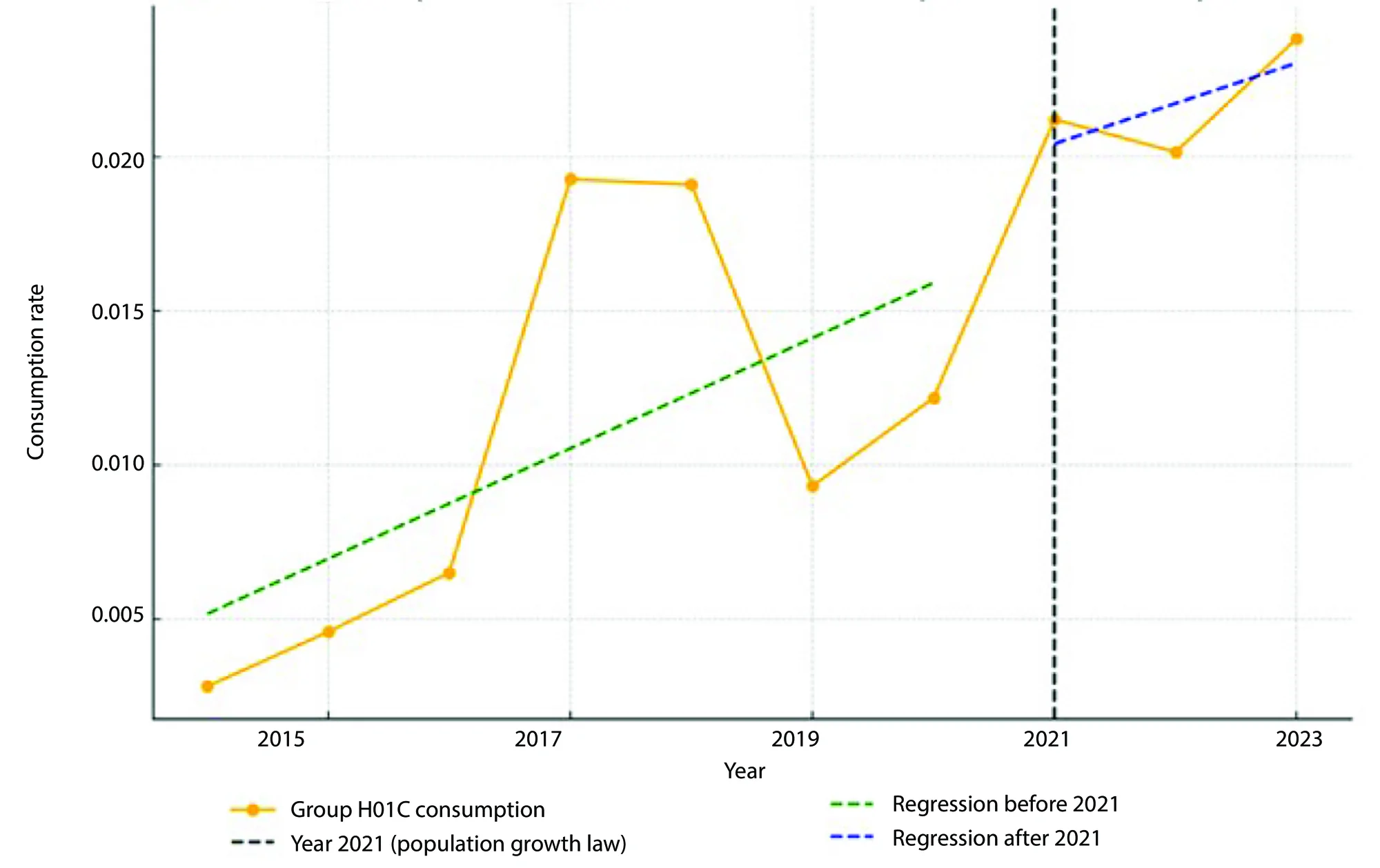
Cetrorelix and ganirelix procurement and consumption trends.

Cetrorelix and ganirelix consumption trends are shown in Figure 5. Overall consumption of these medicines increased during the study period, with a positive slope of +0.0021 that reached statistical significance (P = 0.0044).

### 4.4. Lung Surfactants (ATC R07AA)

**Figure 6.**
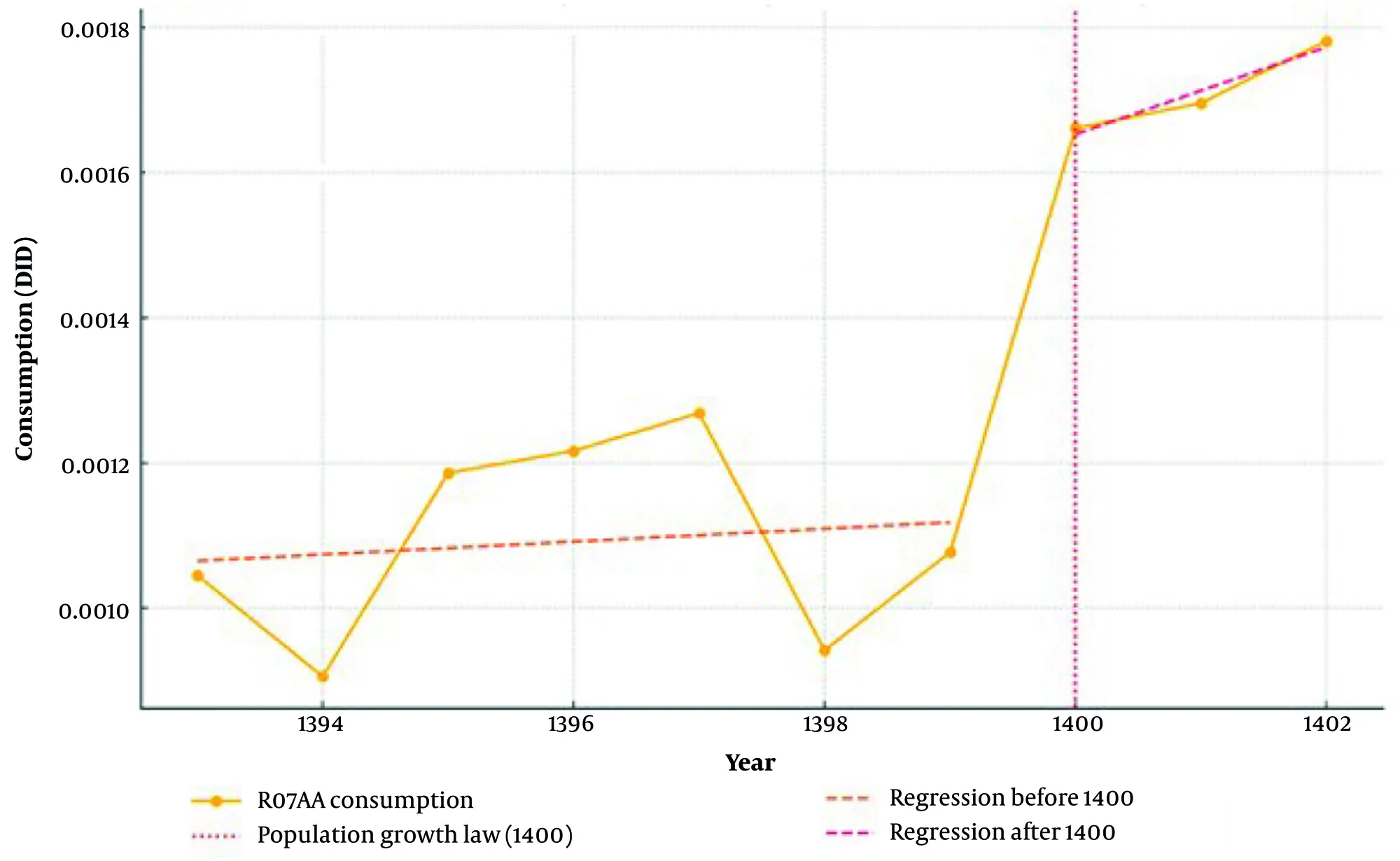
Annual procurement and consumption trends for intratracheal surfactants.

Annual procurement and consumption trends for intratracheal surfactants are shown in Figure 6. Consumption of intratracheal surfactants, including beractant, poractant alfa, and calfactant, more than doubled from 0.012 defined daily doses per 1000 inhabitants per day in 2019 to 0.026 defined daily doses per 1000 inhabitants per day in 2023 (P < 0.001), temporally coinciding with enactment of the law and the COVID-19-related neonatal surge. The postlaw slope increased 5-fold relative to the prelaw baseline (β +0.003 vs +0.0006).

**Figure 7.**
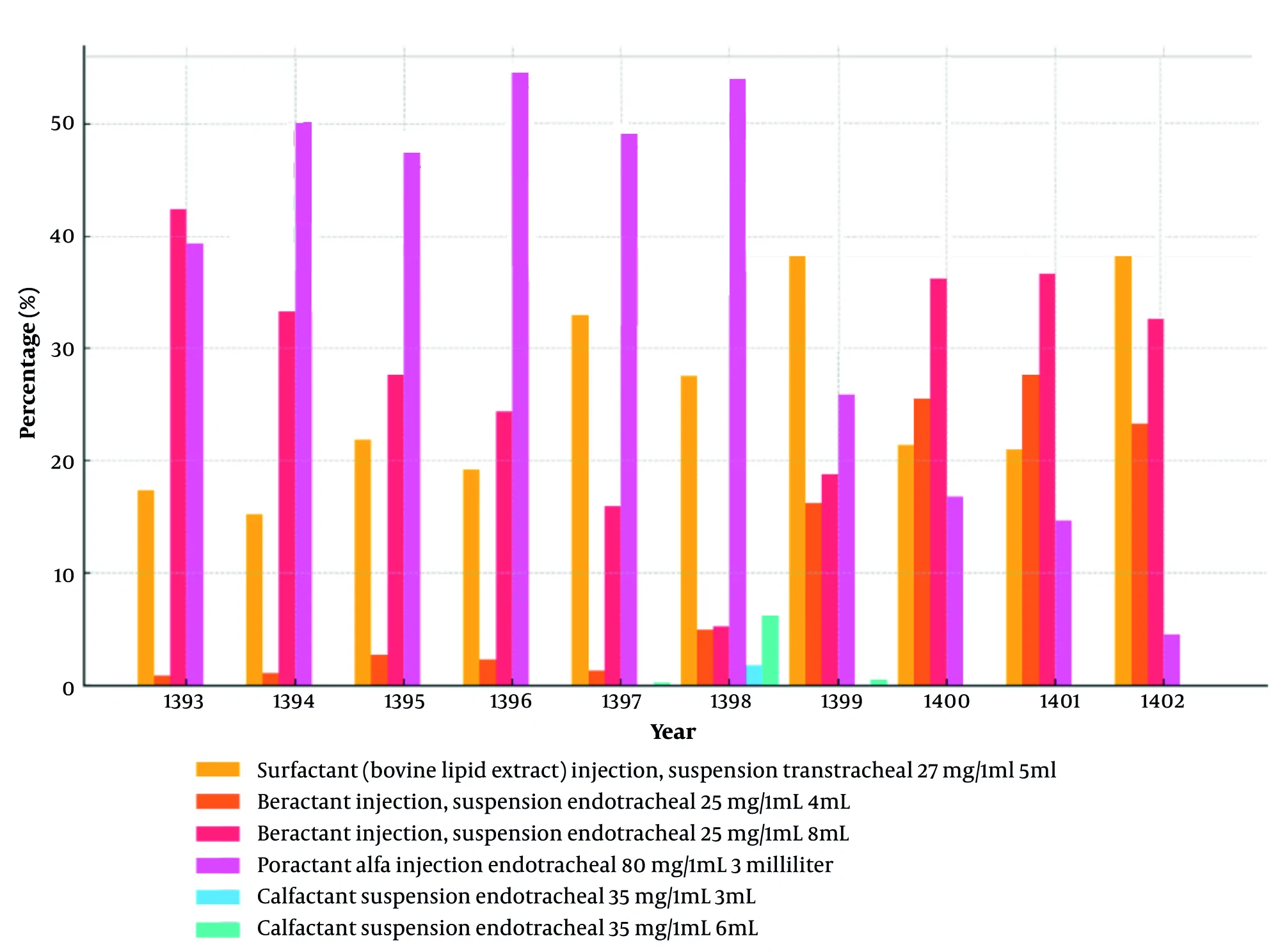
Proportion of each medicine in the total consumption within ATC code R07AA.

The proportion of each medicine in total consumption within the R07AA group is shown in Figure 7.

### 4.5. Medicines Unaffected by the Law

Vitamin K1 injection, oxytocin injection, methylergonovine injection, chlormadinone/ethinyl estradiol tablets, low-dose and high-dose contraceptives, triphasic contraceptives, clomiphene citrate, levonorgestrel emergency pills, lynestrenol, medroxyprogesterone injection, and choriogonadotropin alfa showed neither level nor slope changes. Their collective contribution fell from 41% to 29% of the reproductive-health basket, indicating selective growth in law-favored categories rather than universal expansion.

A summary of selected ATC groups is presented in Table 2.

**Table 2.** Summary of Selected ATC Groups Showing Notable Trends and Prelaw/Postlaw Changes

Row	Medicine/ATC Group	10-Year Trend	Mean Difference After Law	Postlaw Trend Change	P Value	Expected Policy Effect	Observed Outcome
**1**	Prostaglandins (G02AD)	Increasing	Increasing	Shift from increasing to decreasing	NS	Expected decrease	Opposite of expectation (NS)
**2**	Systemic contraceptives (G03A/G03AA/G03AC)	NS	NS	Continuation of decreasing trend	NS	Expected decrease	Not achieved
**3**	Gonadotropins and ovarian stimulants (G03G, G03GA)	NS	Increasing	Shift from decreasing to increasing	Significant	Expected increase	Achieved
**4**	Antigonadotropins (H01CC)	Increasing	Increasing	Mild slowing of increasing trend	NS	Expected increase	Achieved
**5**	Lung surfactants (R07AA)	Increasing	Increasing	Steepening of increasing trend	Significant	No specific expectation	-

## 5. Discussion

The present utilization analysis indicates that the period following the enactment of the Family Support and Population Rejuvenation Law was associated with a heterogeneous pharmaceutical response across Iran’s reproductive-health supply chain.

A methodological limitation of this study is that prescribing data in Iran are fragmented across insurance databases and often exclude paper-based prescriptions. However, wholesale sales data sourced from the Iran Food and Drug Administration provide a continuous and comprehensive proxy. Although these data reflect wholesale distribution rather than direct patient-level consumption, they represent the most reliable source for monitoring national trends and long-term policy impacts in Iran.

The most pronounced increases occurred among infertility-related medicines, including antigonadotrophic agents, menotropins, human chorionic gonadotropin, and recombinant follitropin analogues. These upward shifts were likely influenced by the law’s financial incentives, particularly expanded insurance coverage for assisted-reproductive therapies, which improved affordability and access. This improved access may have primarily benefited individuals already engaged with formal healthcare systems, whereas those with lower health literacy or limited familiarity with specialized reproductive services may have remained disadvantaged (12).

In parallel, the aggregate volume of oral hormonal contraceptives did not decline. Instead, market share shifted from established high-dose levonorgestrel-ethinylestradiol combinations toward newer low-dose desogestrel- and drospirenone-containing regimens. The persistence of these newer medicines suggests that a prescription-only mandate, without accessible counseling or affordable alternatives, does not necessarily suppress demand but instead channels it toward products perceived as safer or more tolerable. Therefore, these findings suggest that the regulatory objective of reducing contraceptive access may not have translated into measurable reductions in overall utilization but rather into therapeutic substitution. This shift likely reflects increasing consumer awareness and knowledge of the side-effect profiles of contraceptive options, rather than a reduction in overall utilization (5).

A finding of particular policy concern is the statistically significant increase in prostaglandin abortifacient agents, notably misoprostol and carboprost. The Act forbids the free or subsidized distribution of these agents, yet their measured sales increased after 2021. Qualitative reports of informal procurement and growth in clandestine online stores reinforce the hypothesis that legal restrictions have displaced demand rather than eliminated it. If so, these trends may indicate a potential shift toward self-managed or informal abortion practices, warranting further epidemiological investigation. Such an outcome conflicts with the statute’s pro-natalist intent and may increase maternal health risks.

Iranian health authorities acknowledge that the rate of abortion in Iran, whether legal or illegal, is high, although exact statistics are unavailable. A substantial proportion of these illegal abortions is believed to involve pharmaceutical agents obtained outside regulated channels (13).

The lack of clinical-level data and outcome verification limits the assessment of dosage accuracy, safety, and complications associated with abortifacient use obtained through informal markets.

The marked increase in neonatal pulmonary surfactant utilization also warrants careful interpretation. Part of this increase may reflect expanded insurance coverage for neonatal care. However, because fetal screening has been limited since implementation of the law, the observed rise in premature-related medication use is concerning. Because data collection coincided with the COVID-19 pandemic, the independent effects of pandemic-related disruptions to maternal care and stress exposure cannot be fully separated from the effects of the policy itself.

Overall, the evidence emphasizes that restrictive measures alone, such as banning subsidized contraceptive methods, are insufficient to modify behavior at the population level and may lead to counterproductive outcomes. A more balanced policy mix is required, coupling sustained financial support for infertility care with culturally sensitive education on family planning and women’s health, the continuous supply of modern contraceptives for those who choose them, and rigorous surveillance of medicine utilization to detect unintended shifts early.

International experience supports this interpretation. Unlike the restrictive pharmaceutical approach applied in Iran, pro-natalist programs in many countries, such as those reviewed by Olivetti and Petrongolo, have largely emphasized supportive interventions rather than limiting access to contraception. These measures commonly include paid maternity leave, parental leave, financial subsidies during pregnancy and postpartum periods, paternity benefits, and improved neonatal care services. Similarly, China’s recent demographic policy shift has focused on creating a more supportive family environment through improved maternal healthcare, affordable childcare, and reduced educational costs, alongside efforts to reshape social norms around marriage and fertility. Family-planning programs influence fertility primarily by expanding access to contraception, improving service quality, and supporting informed reproductive choices rather than through coercive measures. Evidence from Organisation for Economic Co-operation and Development countries similarly shows that effective pro-natalist policies rely on economic and social supports rather than restricting contraceptive or abortion services. In contrast, Iran’s Family Support and Population Rejuvenation Law emphasizes limiting access to certain contraceptive and abortion services alongside financial incentives. Although both approaches aim to modify fertility behavior, international experience suggests that sustainable demographic change is more closely associated with supportive, rights-based interventions than with restrictive approaches (14-16).

Actionable strategies, supported by international evidence and social and economic policies, include expanding community-based reproductive health education, improving access to affordable and confidential reproductive health services, and strengthening regulation and monitoring of pharmaceutical distribution, particularly in informal markets. Evidence suggests that these supportive measures are more effective than restrictive pharmaceutical policies in achieving demographic objectives (12, 14-16).

### 5.1. Conclusions

The law succeeded in its immediate pharmaceutical objective of increasing access to infertility medicines; however, contraceptive utilization did not decline despite regulatory restrictions. Instead, the contraceptive market shifted toward newer low-dose combinations, and prostaglandin utilization increased following implementation of the law, suggesting a potential rise in self-managed or clandestine abortion.

This finding highlights that although financial incentives improved access to infertility care, restrictive measures alone did not reduce the use of abortion medicines and may have unintended public health consequences.

Policymakers should therefore complement financial incentives for childbirth with comprehensive, rights-based family-planning services, strengthened regulation of informal medicine channels, and systematic evaluation of maternal and neonatal outcomes. The findings underscore the necessity of combining financial, educational, and regulatory efforts for effective population policies.

The observed increase in neonatal pulmonary surfactant consumption, occurring alongside limited prenatal screening and broader disruptions related to COVID-19, warrants further investigation. Future studies should specifically evaluate the link between restrictions on prenatal screening and increased neonatal drug utilization to better understand potential health impacts.

Collectively, these findings demonstrate that sustainable demographic progress cannot be achieved through isolated regulatory measures alone. Integrated approaches combining monitoring, evaluation, and supportive policies are critical to inform timely policy refinement and mitigate unintended outcomes.

ijpr-25-1-164664-s001.pdf

## Data Availability

The dataset presented in the study is available on request from the corresponding author during submission or after publication. The data are not publicly available due to privacy and ethical restrictions.
