# Supplementary material for: Impact of Family Planning Program on Related Drug Consumption in Iran: A Ten-Year Trend Analysis (2014 - 2023)
Source: Iran J Pharm Res. 2026 Jun 21;25(1):e164664. doi: 10.5812/ijpr-164664 (PMC13558862; doi:10.5812/ijpr-164664)
Supplement: Supplementary file 1 [file ijpr-25-1-164664-s001.pdf]

Supplementary Table S1. Final List of 52 Medicinal Products Related to the Family Support and Population Rejuvenation Law, Classified by ATC Code.

Medicine	ATC code	1393 DID	1394 DID	1395 DID	1396 DID	1397 DID	1398 DID	1399 DID	1400 DID	1401 DID	1402 DID
Vitamin K 2mg/0.5 mL	B02BA01	0.039	0.048	0.009	0.070	0.056	0.021	0.051	0.028	0.048	0.043
Methylergonovine Maleate Injection Parenteral 0.2 mg/1mL	G02AB03	0.017	0.005	0.016	0.008	0.002	0.002	0.014	0.011	0.022	0.011
Carboprost Injection Intramuscular 250 ug/1mL	G02AD04	0.00000	0.00000	0.00001	0.00001	0.00000	0.00000	0.00000	0.00001	0.00003	0.00002
Misoprostol Tablet Oral 100 ug	G02AD06	0.005	0.007	0.005	0.002	0.003	0.002	0.001	0.000	0.000	0.000
Misoprostol Tablet Oral 200 ug	G02AD06	0.022	0.030	0.017	0.034	0.031	0.032	0.037	0.038	0.042	0.037
Contraceptive HD Tablet Oral 0.25 mg/50 ug	G03AA07	1.475	1.040	1.635	1.156	0.685	0.849	1.865	1.248	1.147	0.632
Contraceptive LD Tablet Oral 30 ug/0.15 mg	G03AA07	20.455	19.117	19.376	17.457	13.092	12.996	12.642	14.010	15.358	13.064
Contraceptive Triphasic Tablet Oral	G03AA07	1.105	1.238	1.095	1.075	0.982	1.231	0.750	0.752	1.228	1.135
Medroxyprogestron/esteradiol 50mg/10mg	G03AA08	0.613	0.495	0.581	0.557	0.761	2.220	1.285	1.495	1.140	1.080
Contraceptive DE Tablet Oral 0.15 mg/20 ug	G03AA09	0.009	0.011	0.038	0.000	0.151	0.176	0.039	0.254	0.107	0.000
Contraceptive DE Tablet Oral 0.15 mg/30 ug	G03AA09	0.712	0.921	0.667	1.086	0.853	1.046	1.091	1.894	1.729	1.707

Drospirenone/Ethynil estradiol 0.02	G03AA12	0.166	0.202	0.285	0.266	0.260	0.122	0.221	0.388	0.462	0.584
Drospirenone/Ethynil estradiol 0.03	G03AA12	1.141	0.921	1.106	1.424	1.138	1.348	1.720	1.620	1.716	2.156
Chlormadinone / Ethinyl Estradiol Tablet Oral 2 mg/0.03 mg	G03AA15	0.002	0.024	0.034	0.042	0.018	0.017	0.000	0.000	0.000	0.009
Dienogest / Ethinylestradiol Tablet Oral 2 mg/0.03 mg	G03AA16	0.000	0.000	0.000	0.000	0.000	0.006	0.002	0.016	0.057	0.068
Lynestrenol Tablet Oral 0.5 mg	G03AC02	0.000	0.716	0.901	0.609	0.000	0.318	0.356	0.294	0.813	0.506
Levonorgestrel Tablet Oral 750 ug	G03AC03	0.127	0.084	0.049	0.062	0.157	0.555	0.387	0.140	0.166	0.160
Levonorgestrel Tablet Oral 1.5 mg	G03AC03	0.158	0.121	0.081	0.174	0.108	0.099	0.128	0.149	0.192	0.167
Medroxyprogestrone 150 mg/mL	G03AC06	0.043	0.031	0.014	0.021	0.009	0.015	0.000	0.014	0.013	0.006
Medroxyprogestrone 150 mg/3.3mL	G03AC06	1.424	2.053	1.599	1.771	1.693	1.798	2.318	3.933	2.693	2.048
Chorionic Gonadotrophin (Human) Injection, Powder, For Solution Parenteral 500 [iU]	G03GA01	0.000	0.000	0.000	0.000	0.000	0.000	0.000	0.000	0.000	0.000
Chorionic Gonadotrophin (Human) Injection, Powder,	G03GA01	0.012	0.022	0.013	0.003	0.003	0.003	0.002	0.005	0.000	0.000

For Solution Parenteral 1500 [iU]											
Chorionic Gonadotrophin (Human) Injection, Powder, For Solution Parenteral 5000 [iU]	G03GA02	1.194	0.973	1.048	0.912	0.779	0.766	0.834	0.777	1.266	1.538
Menotropins Injection, Powder, Lyophilized, For Solution Parenteral 75 [Iu]/75 [Iu]	G03GA02	0.106	0.110	0.047	0.112	0.137	0.090	0.127	0.207	0.247	0.200
Menotropins Injection, Powder, Lyophilized, For Solution Parenteral 150 [Iu]/150 [Iu]	G03GA02	0.000	0.000	0.110	0.002	0.000	0.000	0.000	0.000	0.000	0.000
Urofollitropin Injection, Powder, Lyophilized, For Solution Intramuscular 75 [Iu]	G03GA04	0.004	0.014	0.009	0.011	0.006	0.004	0.004	0.005	0.008	0.007
Follitropin Alfa Injection, Powder, For Solution Parenteral 75 [Iu]	G03GA05	0.045	0.038	0.032	0.032	0.022	0.026	0.022	0.026	0.026	0.027
Follitropin Alfa Injection Parenteral 600 [Iu]/1ml 0.5 Ml	G03GA05	0.002	0.003	0.005	0.003	0.007	0.006	0.007	0.011	0.013	0.013

Follitropin Alfa Injection Parenteral 600 [Iu]/1ml 0.75 Ml	G03GA05	0.002	0.005	0.006	0.007	0.017	0.014	0.016	0.022	0.023	0.023
Follitropin Alfa Injection Parenteral 600 [Iu]/1ml 1.5 Ml	G03GA05	0.002	0.002	0.004	0.020	0.010	0.017	0.030	0.059	0.083	0.089
Follitropin Beta Injection, Solution Parenteral 75 [Iu]/0.5ml	G03GA06	0.0000	0.0000	0.0024	0.0000	0.0035	0.0020	0.0000	0.0000	0.0000	0.0000
Follitropin Beta Injection, Solution Parenteral 833 [Iu]/1ml 0.36milliliter	G03GA06	0.0000	0.0000	0.0000	0.0000	0.0011	0.0014	0.0001	0.0000	0.0000	0.0000
Follitropin Beta Injection, Solution Parenteral 833 [Iu]/1ml 0.72 Ml	G03GA06	0.0000	0.0000	0.0000	0.0000	0.0017	0.0018	0.0012	0.0014	0.0001	0.0016
Follitropin Beta Injection, Solution Parenteral 833 [Iu]/1ml 1.08 Ml	G03GA06	0.0000	0.0000	0.0000	0.0000	0.0009	0.0007	0.0000	0.0013	0.0004	0.0035
Choriogonadotropin Alfa Injection Parenteral 500 Ug/1ml 0.5milliliter	G03GA08	0.001	0.001	0.001	0.001	0.001	0.002	0.002	0.002	0.002	0.001
Corifollitropin Alfa Injection, Solution Parenteral 300 Ug/1ml 0.5milliliter	G03GA09	0.0000	0.0000	0.0000	0.0000	0.0000	0.0001	0.0002	0.0001	0.0001	0.0002
Follitropin Alfa / Lutropin Alfa Injection, Powder, For	G03GA30	0.001	0.001	0.006	0.007	0.009	0.009	0.009	0.006	0.009	0.009

Solution Parenteral 150 [Iu]/75 [Iu]											
Clomiphene Citrate Tablet Oral 50 mg	G03GB02	3.396	2.826	2.661	2.240	1.437	2.072	2.543	1.706	2.223	1.672
Cyproterone Compound Tablet Oral 2 mg/0.035 mg*	G03HB01	1.907	1.975	1.859	1.788	1.645	1.624	1.764	2.303	2.508	2.466
Oxytocin Injection Parenteral 5 [iU]/1mL	H01BB02	0.261	0.281	0.323	0.314	0.171	0.148	0.180	0.223	0.249	0.239
Oxytocin Injection Parenteral 10 [iU]/1mL	H01BB02	0.002	0.001	0.000	0.001	0.000	0.000	0.000	0.000	0.000	0.000
Ganirelix (As Acetate) Injection Parenteral 500 ug/1mL	H01CC01	0.000	0.001	0.002	0.002	0.003	0.002	0.002	0.001	0.003	0.003
Cetrorelix Injection, Powder, For Solution Parenteral 250 ug	H01CC02	0.003	0.003	0.005	0.017	0.016	0.007	0.010	0.020	0.017	0.020
Triptorelin (As Acetate) Injection Parenteral 95.6 ug	L02AE04	0.303	0.323	0.331	0.352	0.286	0.319	0.291	0.592	0.505	0.428
Triptorelin (As Acetate) Injection, Powder, Lyophilized, For Suspension, Extended Release Parenteral 3.75 mg	L02AE04	0.017	0.012	0.020	0.023	0.018	0.031	0.042	0.036	0.014	0.031
Surfactant (Bovine Lipid Extract) Injection, Suspension	R07AA02	0.00018	0.00014	0.00026	0.00023	0.00042	0.00026	0.00041	0.00036	0.00036	0.00070

Transtracheal 27 mg/1ml 5mL											
Beractant Injection, Suspension Endotracheal 25 mg/1ml 4mL	R07AA02	0.00001	0.00001	0.00003	0.00003	0.00002	0.00005	0.00017	0.00042	0.00047	0.00042
Beractant Injection, Suspension Endotracheal 25 mg/1ml 8mL	R07AA02	0.00044	0.00030	0.00033	0.00030	0.00020	0.00005	0.00020	0.00060	0.00062	0.00058
Poractant Alfa Injection Endotracheal 80 mg/1ml 3mL	R07AA02	0.00041	0.00046	0.00057	0.00066	0.00063	0.00051	0.00028	0.00028	0.00025	0.00008
Calfactant Suspension Endotracheal 35 mg/1ml 3mL	R07AA02	0.00000	0.00000	0.00000	0.00000	0.00000	0.00002	0.00000	0.00000	0.00000	0.00000
Calfactant Suspension Endotracheal 35 mg/1ml 6mL	R07AA02					0.00000	0.00006	0.00001			0.00000
